# The effects of an electronic head-mounted display in vision rehabilitation for patients with tunnel vision

**DOI:** 10.1007/s10792-024-02974-5

**Published:** 2024-02-23

**Authors:** Dongye Xu, Manrong Yu, Changyue Zheng, Shunmei Ji, Jinhui Dai

**Affiliations:** 1 National Clinical Research Center for Ocular Diseases, Eye Hospital, Wenzhou Medical University, Wenzhou, 325027 China; 2 Department of Ophthalmology, Zhongshan Hospital Affiliated to Fudan University, No. 180 Fenglin Road, Shanghai, 200032 China; 3 Department of Ophthalmology, Eye and ENT Hospital Affiliated to Fudan University, Shanghai, 200031 China; 4 Department of Ophthalmology, Shanghai Geriatric Medical Center, Shanghai, 201104 China

**Keywords:** Head-mounted devices, Visual aids, Visual field, Tunnel vision

## Abstract

**Purpose:**

To investigate the effect of a new head-mounted electronic visual aid-Acesight on improving visual function and daily activities in patients with tunnel vision.

**Methods:**

57 patients with tunnel vision participated in this study. The visual field (VF), visual acuity (VA), search ability, time of finding people from the side (TFPS), walking ability, and the subjective feelings of patients with and without Acesight were measured.

**Results:**

15 (36%) patients thought Acesight was “helpful”, 16 (28%) thought it was “a little help”, and 26 (46%) believed that it was “not helpful.” The proportion of people aged < 60 years found Acesight helpful was higher. When wearing Acesight, the average horizontal VF diameter (°) (35.54[8.72]) and vertical VF diameter (°) (26.63[5.38]) were larger than those without visual aids (20.61[9.22], 18.19[6.67]) (*P* all < 0.001). The average TFPS before and while wearing the Acesight was 1.77s(0.32) and 1.19s(0.29), respectively (*t *= 14.28, *P* < 0.001). The average search times, number of collisions, walking speeds when wearing the Acesight were not statistically different from those without visual aids (*P* all > 0.05).

**Conclusion:**

More than half of patients with tunnel vision found the Acesight helpful, and a higher proportion of those aged < 60 years old found it helpful. Acesight can expand the horizontal and vertical VF of patients with tunnel vision and can enable patients to detect objects coming from the side earlier.

**Trial registration:**

ChiCTR2000028859; Date of registration: 2020/1/5; URL: http://www.chictr.org.cn/showproj.aspx?proj=47129

## Introduction

Severely restricted peripheral VF (known as tunnel vision) makes some daily tasks extremely difficult, causing collisions, stumbles, and failure to find objects. Tunnel vision will impact patients’ daily functioning, independence, social interactions, quality of life, and mental health. Currently, rehabilitation methods for patients with tunnel vision are still relatively limited. Traditional visual field enlargement aids include reflectors, prisms, [1, 2], and inverted Galilean telescopes [3]. However, these optical visual aids have minimal VF extension, dispersion, imaging bias, and poor patient adaptation, which cannot effectively benefit patients with severe tunnel vision [4–6]. Partial rejection of these devices by tunnel vision patients has been reported, especially in everyday dynamic environments.

In recent years, electronic head-mounted displays (HMDs), a new type of vision aids that integrates cameras, image processing, and displays in a head-mounted device, have been increasingly valued by researchers. This type of vision aids first appeared in the 1990s [7, 8], which improved the visual perception of low-vision patients through a camera with adjustable magnification and autofocus [9, 10]s. Recent advances in camera, display, and computing capabilities have enabled the rapid development of HMDs. Nowadays, HMDs are more convenient and have a broader application range [7, 10]. According to usability, HMDs can be divided into virtual reality (VR, also called immersive reality) devices and see-through displays. VR devices eliminate the direct connection between users’ eyes and the actual environment, and users are immersed in a simulated world. The most common use of see-through displays is augmented reality (AR) applications. It superimposes the computed image directly on the users’ field of view without obstructing it. The advantage of AR devices is that they allow the user to retain his habitual vision while benefiting from the enhanced information of the display. It can maintain the balance of the visual system to a certain extent, reduce the occurrence of motion sickness, and is more suitable for use in sports.

HMD electronic VF enlargement aids may effectively improve the quality of life of patients with tunnel vision. With the advancement of electronic assistive technology, HMDs may offer more possibilities for improving the quality of life of patients with tunnel vision. More studies about HMDs applied to patients with central visual loss [12–14], but fewer applied to peripheral visual field loss (PVL) patients. In 2004, Peli et al. first reported an AR visual aid (LV-3) [9] for patients with tunnel vision, which superimposes the reduced contour information of a large field on the patient’s central VF without blocking the original field of view. Since then, few clinical applications of this visual aid have been reported. This study aimed to evaluate the clinical application effects of a new AR head-mounted visual aid—Acesight—on the visual function and daily activity ability of patients with tunnel vision.

## Methods

### Patients and methods

Participants were selected from patients attending the Low Vision Clinic of the Eye and ENT Hospital of Fudan University from October 2019 to September 2021. To be included in the study, the patients had to meet the following inclusion criteria: each patient had received formal treatment for the primary disease, and the eye condition remained stable for at least six months; patients were confirmed to have PVL by visual field examination, and the residual VF diameter ≤ 40°; each patient could voluntarily try the visual aids and have no obvious language or intellectual impairment. The patients were divided into two groups: < 20°and ≥ 20° by mean visual diameter for analysis. This study followed the tenets of the Declaration of Helsinki and was approved by the Ethics Committee of the Eye and ENT Hospital of Fudan University. All participants signed informed consent forms.

### Acesight vision aid

**Fig. 1:**
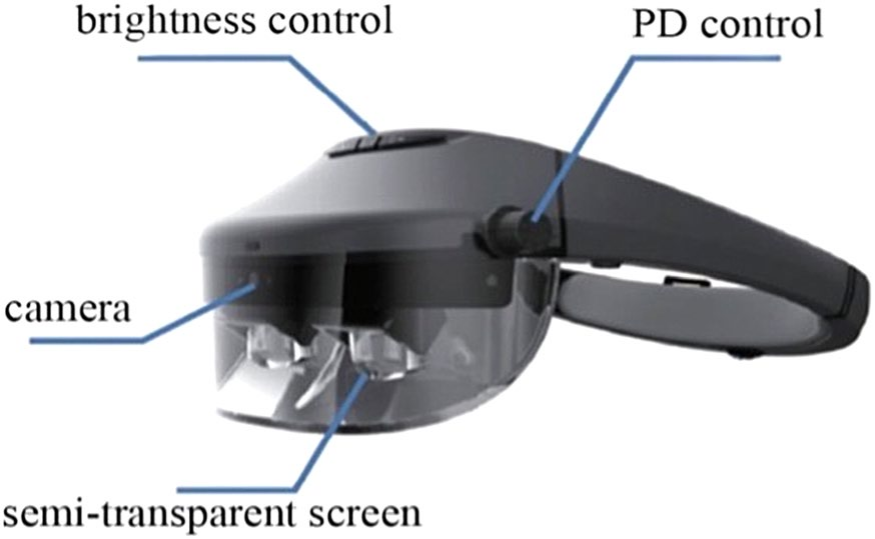
Appearance and modules of Acesight

The new head-mounted aid used in this study was Acesight AR (www.rejointech.com.cn) (Fig. 1). The display uses a free-form AR display module, superimposing the natural environment and the computed picture in real time to the same space through a semi-transparent screen. It has an eight million autofocus camera and a six-axis positioning sensor. Its picture field diameter is 90°, with a resolution of 1080P and magnification of 0.25 ~ 9x. It has 10 color-changing modes and three contour enhancement modes. The patient controls the display mode through a remote control. When applied to patients with PVL, it can reduce the picture by 0.5 or 0.25 times and compresses information from a wide visual field into the remaining narrow visual field of the patients. Because it adopts a semi-transparent display, the patient’s field of vision outside the display screen is not affected, and they can still observe the surroundings and objects below by rolling their eyes. In addition, according to the visual problems of different people, it controls the image zoom and displays the contrast and discoloration effect of the projected picture through different intelligent algorithms.

Before the experiment began, the concepts of Acesight were explained, and the subjects received a training session. During the test, the patient can freely change the magnification of the visual aid to obtain the best vision effect. The display mode defaulted to the “color mode.” If the subject thought that a particular image gain mode helped improve visual impact, then the test was done in that gain mode.

### Measurements

All the subjects’ sociodemographic characteristics (gender, age, educational level, occupation [working or not], primary disease, and symptom duration) were recorded. Educational status was divided into three levels: primary education (primary school or below), secondary education (junior high school, high school, or secondary school degree), and higher education (college degree, bachelor’s degree, or above).

Acesight allowed patients to correct their refractive errors by wearing spectacles. The binocular distant visual acuity of the participants was measured using a standard logarithmic visual acuity chart (working distance of 5 m), with their spectacles, and with both spectacles and Acesight. For statistical analysis, visual acuity (VA) was recorded as a logarithm of the minimum angle of resolution (logMAR) value.

**Fig. 2:**
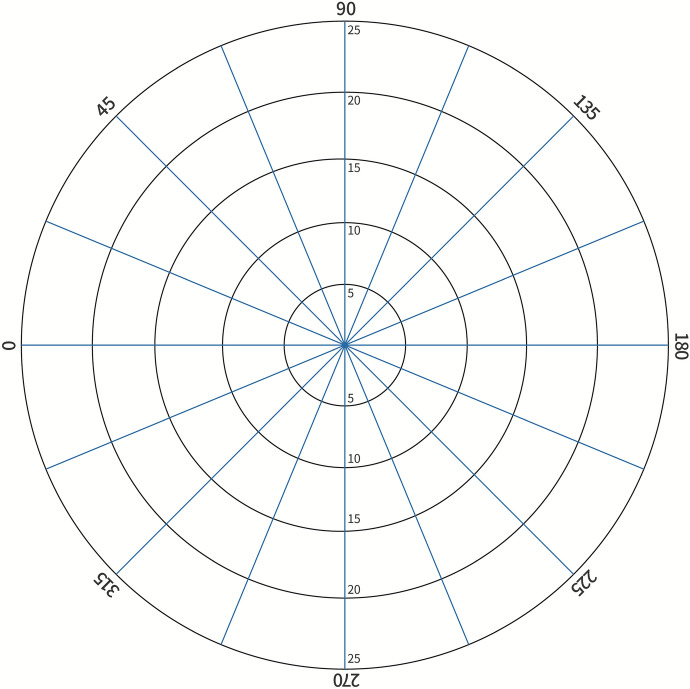
Tangent screen for VF test

Binocular VF was measured using a tangent screen with a 20 mm target from 1 m under standard office illumination (400 lx), which is a dynamic visual field detection method (Fig. 2). It is a more convenient and feasible method in the case of wearing Acesight. Repeat three times in each direction to obtain the average value. Calculate the visual field diameter according to the distance in the horizontal and vertical directions and the distance between the patient and the screen and record it in terms of angle. We also confirmed that all subjects had a single patch of central residual VF (Goldmann V4e). The horizontal and vertical field diameters of each patient without visual aid and with Acesight were detected, and the average VF diameter (the average of the horizontal and vertical diameters) was calculated.

In this study, the independent walking speed of each patient was tested without visual aids and with Acesight. The patients walked the same distance (10 m) independently in an indoor environment with three obstacles on the path, such as stools and signs, etc. The illuminance level was about 200lx. The subjects were instructed to walk to the far end of each corridor (white walls and gray floor), avoiding contact with all obstacles and the corridor walls. The obstacle’s position was different each time to prevent interference with the patient’s memory. The time taken to complete the journey and the number of collisions were recorded. The experiment was carried out in the company of an investigator to ensure patients’ safety.

To test each patient’s search ability, the researcher randomly placed ten various items on a white table (illuminance at the table about 400 lx), asked the patient to find an object and recorded the time to find the item. The subjects were allowed to move their eyes and heads freely during the search. The contents and positions of the items on the table differed for each experiment. The experiment was repeated three times to obtain the average. It was performed when the patient was not wearing a vision aid and when the patient was wearing Acesight.

Patients with tunnel vision usually cannot detect people or vehicles coming around in time, which leads to danger. In this study, the application of Acesight to this problem was simulated. The researcher approached the patient from 1 m in front at 0.5 m/s from 1 m from the side. We recorded the time when the patient noticed the researcher. The time of finding people from the side (TFPS) when patients were not wearing vision aids and when they were wearing Acesight was recorded.

After each patient had learned to use Acesight, we asked them to answer the following questions:

1. Do you think Acesight is helpful to you?
  A: Not helpful; B: A little help; C: Helpful.
2. If it is helpful, what is the main help?
3. If it is not helpful, what is the reason for not helping?
4. What do you think Acesight needs to improve?

The responses of each patient were recorded and summarized for analysis.

### Statistical analysis

IBM SPSS version 24.0 was used for the statistical analysis of the data. GraphPad Prism 8 was used to create figures. The fit of the numeric measurements to normal distribution assumptions was tested using the Shapiro–Wilk test. Continuous measurement data were described as mean (SD). A paired t test was used to compare the VA, VF, walking ability, search ability, and TFPS with and without Acesight. The proportion of different populations that thought Acesight was effective was tested using the Kruskal–Wallis test. A significance level of 0.05 was used in all tests.

## Results 

A total of 57 patients were included in the study. The clinical and demographic characteristics of the included subjects are summarized in Table 1. The average horizontal VF diameter of the included patients was 20.16°, and the average vertical VF diameter was 18.19°. The causes of tunnel vision were glaucoma in 43 (75%) patients and retinitis pigmentosa in 14 (25%) patients.

**Table 1:** Characteristics of the included subjects

Demographics	Tunnel vision (*n* = 52)
Age (y)	58.30(13.26) (18–86)
Gender (Male)	28(49%)
Educational status	
Primary education	20(35%)
Secondary education	20(35%)
Higher education	17(30%)
Symptom duration (y)	8.39(13.23) (1.00 to 50.00)
VA (logMAR)	00.62(0.50) (0.00–2.00)
Horizontal VF diameter (°)	20.61(9.22) (5.00–40.00)
Vertical VF diameter (°)	18.19(6.67) (6.00–35.00)

**Fig. 3:**
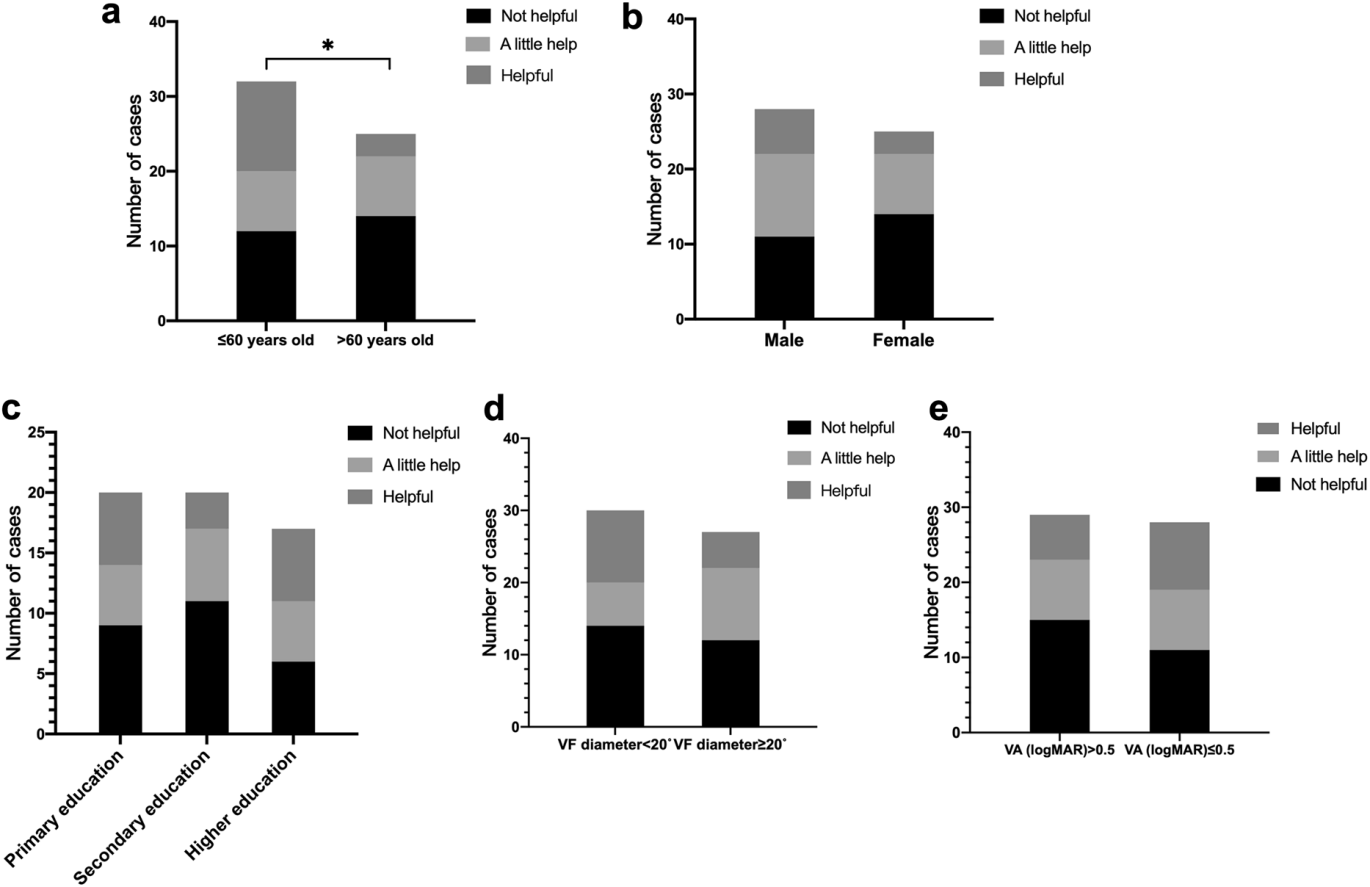
Subjective feelings about Acesight of patients with tunnel vision **a** Subjective feelings of patients of different age groups. **b** Subjective feelings of patients of different gender. **c** Subjective feelings of patients of different educational status. **d** Subjective feelings of patients of different VF diameter groups. **e** Subjective feelings of patients of different VA groups

After learning to use Acesight and wearing it for several hours, we asked them to answer the question, “Do you think the Acesight is helpful to you?” Among 57 patients, 15 (36%) patients chose “Helpful,” 16 (28%) patients chose “A little help,” and 26 (46%) patients chose “Not helpful.” The proportion of patients who were < 60 years old who chose “Helpful” was higher (*P* = 0.04) (Fig. 3).

Among the 31 patients who chose “A little help” or “Helpful,” 13 patients (42%) believed that Acesight increased brightness when the outside light was dim, 11 patients (35%) thought that Acesight could help to walk independently, ten patients (32%) believed that Acesight could help to see distant objects, and six patients (19%) believed that Acesight could help to search objects. The reasons why patients think that Acesight was not helpful included VA decrease in VF enlargement mode (6 patients), inability to judge the distance of objects (six patients), motion sickness (five patients), visual fatigue (four patients), visual field not being large enough (three patients), heavy (two patients), etc. The areas patients thought must be improved about Acesight included improving display clarity, improving portability, redesigning it to look more like regular glasses, expanding the camera range, adding an antishock function to avoid dizziness, etc.

**Fig. 4:**
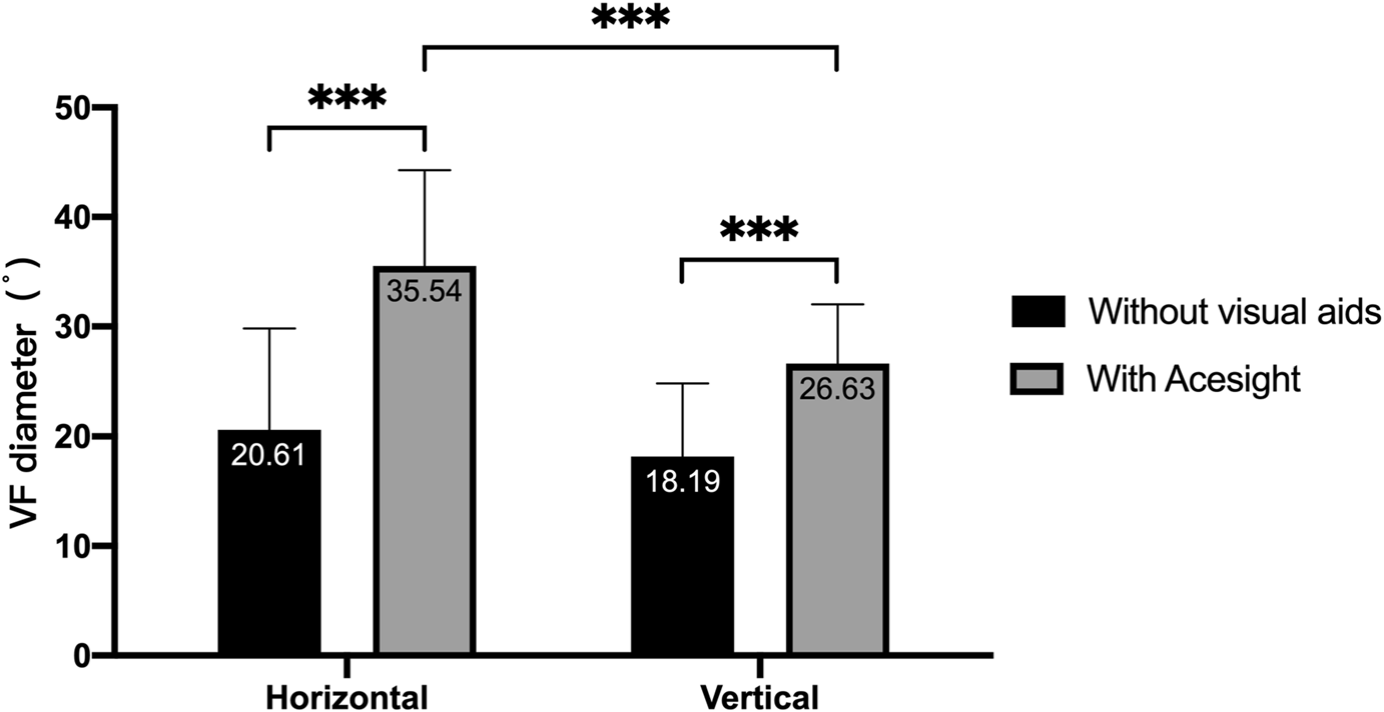
VF diameter of patients with tunnel vision with and without Acesight

When the patients wore Acesight, the average horizontal VF diameter and vertical VF diameter were significantly enlarged (Fig. 4). There was no statistical difference between the horizontal and vertical VF diameters without Acesight (Fig. 4). When wearing Acesight, there was a statistical difference between the horizontal and vertical VF diameters (*t* = 9.28, *P* < 0.001). The horizontal diameter enlargement (14.93° [6.87]) was greater than the vertical diameter enlargement (8.44° [5.01]), with a statistically significant difference (*t* = 8.25, *P* < 0.001). The VA of patients with tunnel vision before and after wearing Acesight (expansion mode) was compared. The results showed that there was a statistically significant difference between the VA with (0.89[0.53]) and without Acesight (0.62[0.50]) (*P* < 0.001).

**Fig. 5:**
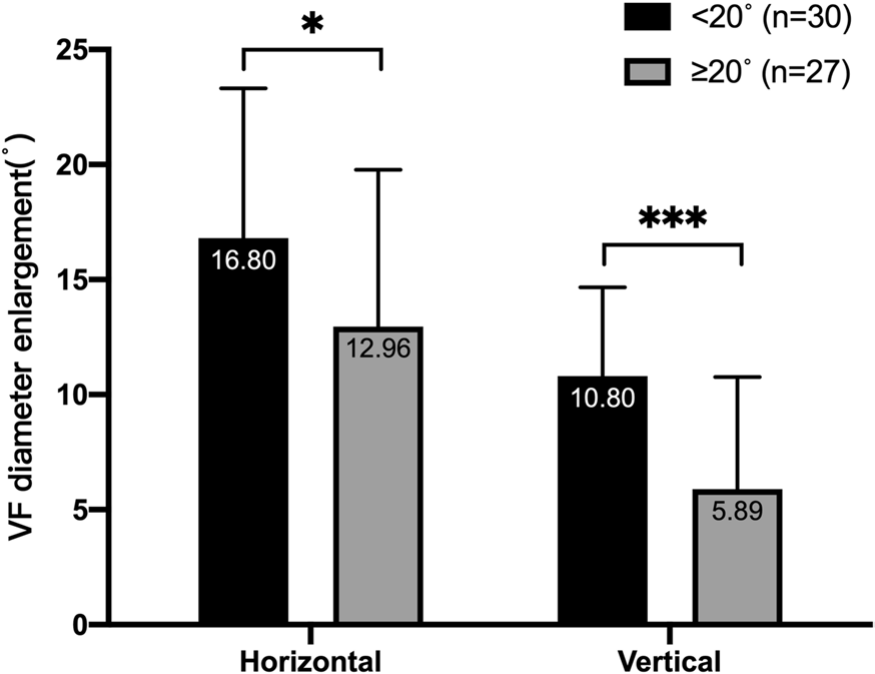
Horizontal and vertical VF diameter enlargement in < 20°group and ≥ 20°group

The patients with tunnel vision were divided into two groups: < 20° (*n* = 30) and ≥ 20° (*n* = 27) by mean visual diameter, and the changes in the horizontal VF diameter and vertical VF diameter with and without Acesight were compared. The results show that the difference between the horizontal VF diameter and the vertical VF diameter enlargement of the < 20°group was greater than those in the ≥ 20°group (*P* = 0.03, *P* < 0.001) (Fig. 5).

The ability of daily activities of patients with tunnel vision with and without Acesight was compared and analyzed (Table 2). The mean TFPS was shortened, and there was a statistically significant difference. The average search time was slightly shorter when wearing Acesight, and there was no statistically significant difference. There was no statistically significant difference between the average number of collisions with and without Acesight. The average walking speed was slower when wearing Acesight, and there was a statistically significant difference.

**Table 2:** The ability of patients with tunnel vision to perform daily activities with and without Acesight

	Without visual aids	Wearing Acesight	*t*	*P*
TPFS (s)	1.77(0.32)	1.19(0.29)	14.28	< 0.001
Search time (s)	1.89(0.84)	1.81(1.13)	0.59	0.56
Walking speed (m/s)	1.76(0.20)	1.07(0.28)	11.17	< 0.001
Number of collisions	0.40(0.61)	0.56(0.71)	− 1.53	0.13

## Discussion

Currently, rehabilitation methods for patients with tunnel vision are still relatively limited. Existing VF expansion aids include reflectors, prisms, inverted Galilean telescopes, etc. However, the expansion of VF of these aids is minimal, and there are shortcomings, such as dispersion, imaging deviation, and poor patient adaptation [4–6]. HMDs avoid these problems. They can significantly expand VF and adjust the display mode according to the needs of different patients, which is expected to provide adequate assistance for patients with severe tunnel vision [11–13]. There are several augmented reality devices available on the market, including eSight. Studies have shown that eSight can improve visual function and increase the quality of life of patients with central vision loss [14, 15]. However, there are few studies of HMD applied to patients with PVL (especially tunnel vision), and there is a lack of research about the effect applied in the activities in real environments. In addition, the previously reported VF expansion HMD visual aids all used the contour display mode. The Acesight AR studied in this paper is a new head-mounted visual aid that uses a reduced image display mode, which can provide more information than a contour. To the best of our knowledge, no studies have reported the clinical application effect of this type of visual aids mode.

More than half (54%) of the 57 patients in this study found Acesight helpful. Young-to middle-aged patients were likelier to learn and understand the use patterns of the visual aids and thought it useful. Other studies have found that young patients were more receptive to VR HMD visual aids [16, 17], and this study demonstrates that younger patients were more receptive to AR HMD visual aids as well.

In this study, Acesight significantly enlarged the horizontal and vertical VF of patients with tunnel vision. In previous research, HMD visual aids can expand the VF to a radius of 10° to 15° [18, 19]. The horizontal VF diameter in this study was expanded to about 35°, and the vertical VF diameter was extended to about 30°, which was better than in the previous study. In this study, the expansion of the horizontal VF diameter was greater than the vertical VF. This may be because the VF angle of Acesight image acquisition is 25° radius in the horizontal direction and 15° in the vertical direction, and the horizontal direction is larger than the vertical direction. In addition, our study showed that Acesight was more effective at expanding VF in patients with a VF diameter of < 20° than in patients with a VF diameter of ≥ 20°. This indicates that Acesight has a better effect on VF enlargement for patients with more severe VF damage.

Patients with tunnel vision are prone to danger due to narrowed VF and difficulty noticing pedestrians or vehicles passing on the side of the walking path. In addition, the patient’s mobility can be reduced because of a reduced ability to spot obstacles, resulting in navigation difficulties. In this study, after wearing Acesight, patients could detect people moving sideways earlier and avoid the risk of collision to some extent.

The HMD vision aids reported by Peli et al.[20] for patients with PVL compressed the contour information of the larger field of view and superimposed it in the patient’s central field of view. Compared with no enhancement, minification with contour images also improved visual search performance when the participants’ original VF was not too limited, and auditory cues decreased search time for all the participants by 54% on average [21]. However, the visual field search time was prolonged in patients with severe tunnel vision (field diameter < 10°) [22]. Acesight uses AR display technology in this study to compress information from a wide visual field into the patient’s residual visual field. There was no significant difference in the search ability of patients with tunnel vision with and without Acesight. These different results may be due to the different display modes and testing methods of visual aids. The technique used by Peli et al. was to find a particular low-contrast letter on a white screen, while our study was in a real environment. In addition, due to the small number of participants included (12 in the former study and 57 in this study), the study’s results were susceptible to the influence of the participants. Patients with tunnel vision mainly search for objects by rolling their eyes and turning their heads. In addition to expanding the VF, adding contours to objects and improving contrast could theoretically help in searching. Which display mode is more helpful in improving the search ability of patients with tunnel vision needs further study.

Our study found that Acesight may reduce the speed of independent walking in patients with tunnel vision in the first use. Similar results have been found in previous studies of other VF enlargement aids [12, 23]. When wearing an HMD, patients were more hesitant to walk independently, thus decreasing their walking speed. VF expansion strategies include compressing visual information, image superposition, etc. Still, these strategies will lead to changes in the patient’s visual perception mode, and it is often difficult for patients to adapt quickly to the new visual perception mode in first use. Acesight compressed visual information into the patient’s residual VF in this study. At the same time, the reduction of the image would change the spatial perception, and the distance perception of patients would worsen. In addition, patients with tunnel vision often compensate for visual field loss by rolling their eyes. When patients wear HMDs, they need to turn their heads to change their field of vision, which is different from how they are accustomed to it. The reduction in mobility ability may relate to the limitations imposed on the field that can be scanned by eye movements alone (without head movement) when wearing the device. Due to the minification factor, training may be necessary to gain a veridical perception of visual direction and correspondence between the real world and the displayed image. Other studies have shown that a period of rehabilitation guidance and training may help improve the acceptance of HMD visual aids [24, 25]. Further study is needed to determine whether the effect will be enhanced after a more extended period of training and use of the device in realistic outdoor settings.

Our study found that VA was decreased with the Acesight device in VF expansion mode compared to when not worn, due to the reduction of the image. When patients with tunnel vision wear Acesight during movement, the VF expansion mode can help detect people and vehicles on the side earlier, reducing the risk of collision. According to our study, more patients with VA (logMAR) ≤ 0.5 thought the Acesight helpful (Fig. 3e). We thought that the VF expansion mode is more suitable for patients with better VA, which is necessary to process the reduced image display. When the patient uses it in static scenarios, they can switch to magnification mode, and the Acesight will display an enlarged image so that the patient can see the details clearly and avoid the inconvenience caused by vision loss.

Subjects were allowed to comment freely on Acesight and how they thought it could be improved. Despite the open-ended nature of the question, several common themes emerged. The improvement directions of HMD VF expansion aids in the future include: (1) Improve clarity to expand VF while minimizing the loss of clarity; (2) Improve portability, such as adopting a glasses-like appearance, which is convenient for patients to use outdoors; (3) Expand the camera range and further improve the ability of VF expansion; (4) Increase the anti-shake function to reduce image jitter caused by autofocus during head movement and avoid dizziness, fatigue, and other problems caused by it.

There were several limitations to this study. The tests in our study were all performed without the device first and then with the device. Several hours of training between tests may have diminished the memory effect, but it could not be avoided altogether. In this study, patients only adapted to Acesight for several hours, and some patients’ walking ability was improved after training. Further study is needed to determine whether the effect will be enhanced after a more extended period of training and use of the device in realistic outdoor settings.

## Conclusion

This study confirmed that HMD visual aids using the image reduction mode could expand the VF of patients with tunnel vision so that patients can detect pedestrians coming sideways earlier. In the first use, patients’ function in some dynamic scenarios did not improve. Under the circumstance that there were currently no effective measures to improve the quality of life of patients with tunnel vision, the augmented-view concept could be a valuable VF expansion aid for obstacle avoidance and hazard prevention.
